# Study on Gesture Recognition Method with Two-Stream Residual Network Fusing sEMG Signals and Acceleration Signals

**DOI:** 10.3390/s24092702

**Published:** 2024-04-24

**Authors:** Zhigang Hu, Shen Wang, Cuisi Ou, Aoru Ge, Xiangpan Li

**Affiliations:** 1 School of Medical Technology and Engineering, Henan University of Science and Technology, Luoyang 471023, China; hu.robert@163.com (Z.H.); tomatocsi@163.com (C.O.); 220320221769@stu.haust.edu.cn (A.G.); 2 School of Mechanical and Electrical Engineering, Henan University of Science and Technology, Luoyang 471003, China; xiangpanli@haust.edu.cn

**Keywords:** human–computer interaction, gesture recognition, two-stream residual network, multi-source information fusion, attention mechanism

## Abstract

Currently, surface EMG signals have a wide range of applications in human–computer interaction systems. However, selecting features for gesture recognition models based on traditional machine learning can be challenging and may not yield satisfactory results. Considering the strong nonlinear generalization ability of neural networks, this paper proposes a two-stream residual network model with an attention mechanism for gesture recognition. One branch processes surface EMG signals, while the other processes hand acceleration signals. Segmented networks are utilized to fully extract the physiological and kinematic features of the hand. To enhance the model’s capacity to learn crucial information, we introduce an attention mechanism after global average pooling. This mechanism strengthens relevant features and weakens irrelevant ones. Finally, the deep features obtained from the two branches of learning are fused to further improve the accuracy of multi-gesture recognition. The experiments conducted on the NinaPro DB2 public dataset resulted in a recognition accuracy of 88.25% for 49 gestures. This demonstrates that our network model can effectively capture gesture features, enhancing accuracy and robustness across various gestures. This approach to multi-source information fusion is expected to provide more accurate and real-time commands for exoskeleton robots and myoelectric prosthetic control systems, thereby enhancing the user experience and the naturalness of robot operation.

## 1. Introduction

Surface electromyography (sEMG) is a bioelectrical signal that records muscle activity [1]. It provides rapid information about muscle contraction and relaxation by attaching electrodes to the muscle surface. Due to its non-invasive and real-time nature, myoelectric signal-based gesture recognition technology has gained significant attention in fields such as rehabilitation medicine [2], sign language recognition [3,4], handwriting recognition [5], intelligent prosthetics [6], and exoskeleton robots [7]. In comparison to methods like kinematic analysis, surface electromyography (sEMG) signals allow for the efficient and accurate recognition of gestures by accessing the co-activation of muscles. This facilitates natural and convenient human–computer interaction in areas such as rehabilitation exoskeleton robots and prosthetic limb control [8,9,10].

The predominant method for electromyographic gesture recognition involves capturing surface electromyography signals, extracting pertinent features, and subsequently employing a classifier to map these features to respective gesture categories [11]. Typically, time-domain features (such as mean, variance, and time-domain waveform), frequency-domain features (such as power spectral density), and time–frequency-domain features (such as wavelet transform coefficients) are used as features [12]. Researchers usually extract multiple features and then utilize a feature optimizer to process the feature set. Classifiers commonly employed include support vector machines (SVM) [13], k-nearest neighbors (k-NN) [14], artificial neural networks (ANN) [15], and other machine learning methods. There is no universal model for all application requirements and datasets; therefore, most studies have attempted various algorithms [16] to determine an appropriate one based on their performance and other requirements.

In recent years, there has been significant development in the field of gesture recognition based on deep learning. Deep neural networks perform well in image processing and can effectively extract image features, resulting in significant progress in target detection and gesture interaction for computer vision-based gesture recognition [17,18]. On the other hand, bioelectrical signal-based gesture recognition has also gained attention. Deep learning models can effectively learn the mapping between biosignals and gesture actions. This trend presents new opportunities and challenges for gesture recognition technology [19]. Commonly used deep learning models include convolutional neural networks (CNNs), recurrent neural networks (RNNs), and their variants. In their exploration of the field of gesture recognition based on bioelectrical signals, Atzori et al. [20] utilized CNNs for the first time on the publicly available dataset NinaPro DB2 to classify 49 gestures with an average accuracy of 60.27 ± 7.7%. Geng et al. [21] proposed a transient-based EMG image-based CNN architecture that improved the average accuracy of 49 gestures to 76.1% on the NinaPro DB2 dataset. Ding et al. [22] proposed a parallel multiscale convolutional structure with an average accuracy of 78.86% for 17 gestures on the NinaPro DB2 dataset. Deep learning models can automatically learn and extract features from data, reducing the need for manual feature extraction and often achieving better recognition performance.

The classification of gestures based on EMG signals is commonly viewed as a pattern classification issue. At present, there are two main methods for classifying gestures: sparse multi-channel sEMG-based methods and high-density sEMG-based methods. Sparse multi-channel sEMG methods typically employ a small number of surface EMG signal sensors to capture muscle electrical activity. These methods typically extract time-domain or frequency-domain features for input into traditional classifiers. HD-sEMG methods, on the other hand, employ more sophisticated feature extraction methods to capture richer information about muscle activity [23,24]. Deep learning models are better able to handle high-dimensional and complex data, leading to a higher level of gesture recognition and muscle control.

## 2. Materials and Methods

### 2.1. NinaPro Dataset

**Figure 1:**
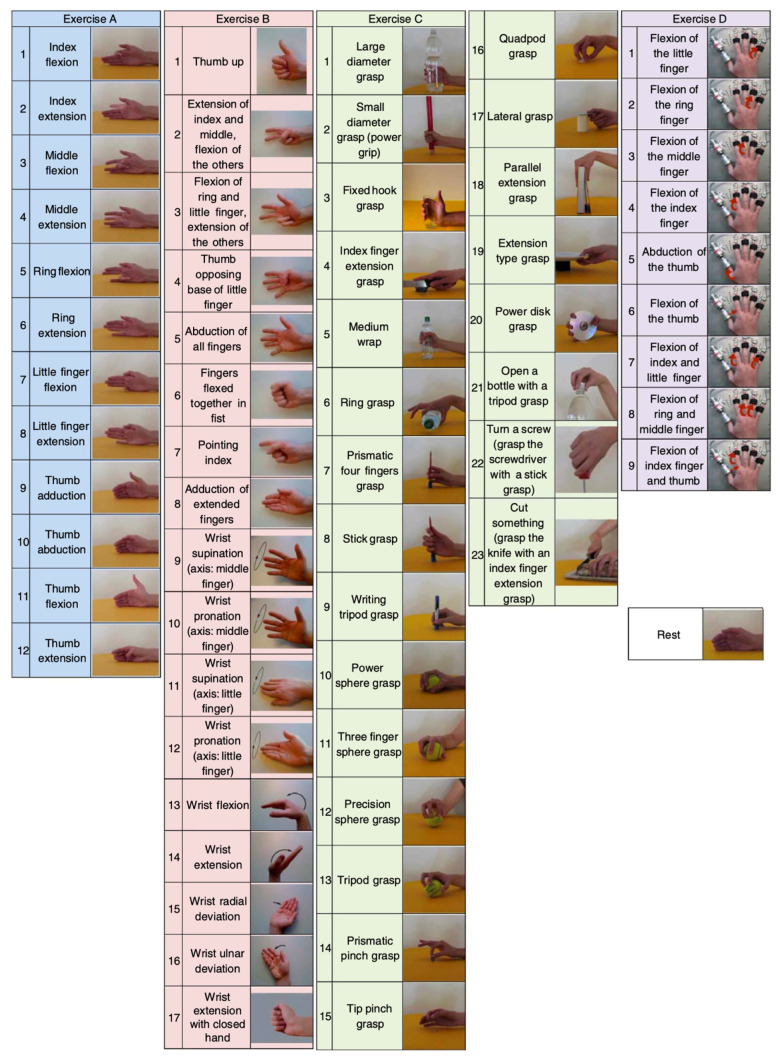
NinaPro dataset 49 categories of gesture actions.

The study utilized the DB2 dataset from the NinaPro database [25] as the source of experimental data. The NinaPro database is a publicly available multimodal database created to facilitate research and development in the myoelectric control community. The DB2 dataset is a subset of the NinaPro database. It contains EMG, inertia, kinematic, and force data from 40 intact subjects during 49 repetitions of hand movements plus rest. The grouping of the 50 labels corresponding to the gesture categories is shown in Table 1:

In this case, each type of gesture movement was repeated six times, each lasting for five seconds, followed by a three-second rest. The sEMG data for gesture movements were acquired using 12 Trigno wireless electrodes at a sampling rate of 2000 Hz. Each electrode was equipped with a triaxial accelerometer. For each movement, 12 columns of EMG signals and 36 columns of acceleration signals related to the hand were recorded. The initial eight electrodes were placed equidistantly at the radial humeral joint of the forearm. The 9th and 10th electrodes recorded signals from the primary activity points of the superficial finger flexors and extensors, while the 11th and 12th electrodes recorded signals from the primary activity points of the biceps and triceps muscles. Figure 1 displays the gesture movements.

For the division of the dataset, 2/3 of the movement repetitions (Repetitions 1, 3, 4, and 6) of each subject were used as the training dataset, and the remaining 1/3 of the movement repetitions (Repetitions 2 and 5) was used as the test dataset [26,27]. During the training process, 1/10 of the training set was classified as the verification set. The hyperparameters of the model were adjusted by verifying the set to avoid over-fitting and to find the optimal network structure. The accuracy of classifying a single gesture was determined by the ratio of correctly classified gesture segments to the total number of segments tested in a trial. The accuracy (*Acc*) for a single target object was calculated as follows:

(1) $$Acc=\frac{Number\;of\;correct\;classifications}{Total\;number\;of\;test\;samples}$$

The overall accuracy (*OA*) of classification was calculated as the average accuracy of gesture recognition in the test set of *N* subjects, which was calculated as shown in the following equation:

(2) $$OA=\frac{1}{N}\sum_{N}\frac{Number\;of\;correct\;classifications}{Total\;number\;of\;test\;samples}=\frac{1}{N}\sum_{N}Acc$$

### 2.2. Data Analysis and Processing

**Figure 2:**
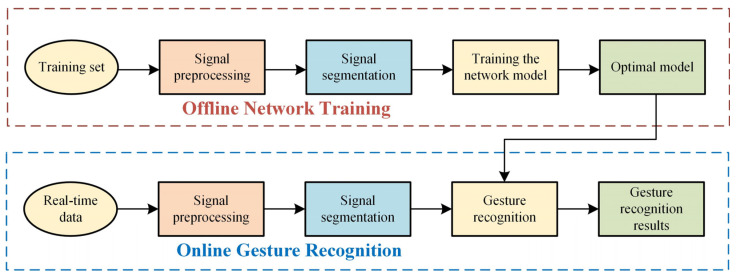
Hand gesture recognition processing flow.

The overall framework flow of the gesture recognition method is shown in Figure 2. Experiments were conducted in this paper using data from the Ninapro DB2 dataset. The data of each subject were preprocessed to fit the inputs of the neural network model. The preprocessing stage involved filtering and noise reduction, normalization, and then intercepting the processed surface EMG and acceleration signals to divide the training and test sets. The training set was fed into the network in batches to iteratively train a model with high classification accuracy. Simultaneously, the optimal model was used to validate the sample data from the test set. The validated model predicts real-time input data, classifies gestures, and facilitates related control and decision-making.

### 2.3. Preprocessing

As a type of physiological electrical signal, the pre-processing of EMG signals is necessary when processing them [26]. sEMG signals are characterized by their small amplitude (mV), high noise, and non-stationarity, making EMG signals difficult to analyze. Acceleration signals can be influenced by environmental factors such as vibration, movement, or other disturbances from the device. Preprocessing was performed on both signals. This included filtering and denoising, Z-score normalization, and signal segmentation. These preprocessing steps improved the reliability and accuracy of the subsequent analysis by bringing out the true characteristics of the signal.

#### 2.3.1. Filtering and Denoising

The surface electromyography (sEMG) data in NinaPro DB2 were processed to remove any interference from the power line frequency (IF) and baseline offset. The useful signal energy of sEMG data is primarily distributed between 10 Hz and 500 Hz [28,29]. To maintain the original characteristics of EMG and acceleration signals, this paper utilized a fourth-order Butterworth filter with a passband boundary of 10~500 Hz for the bandpass filtering of the two signals.

#### 2.3.2. Normalization

The Ninapro DB2 database comprises EMG and acceleration signals obtained from various individuals [25]. These signals were acquired from a wide range of hand muscle activities, and their amplitude ranges were typically in the microvolt range (μF). To account for individual differences, as well as differences in the intensity and quality of muscle activity, there may be significant variations in the signal amplitudes. Z-score normalization is a widely used method in signal processing and modeling to address amplitude differences. This method scales the signal’s amplitude to a standard normal distribution with a mean of 0 and a standard deviation of 1. Through this normalization process, amplitude differences can be eliminated to improve the consistency and reliability of the data. This aids in accurately understanding the characteristics of the experimental data and enhancing the effectiveness of signal processing and modeling, thereby improving the performance and stability of the gesture recognition system [30].

Before training, the data in the training set were subjected to a Z-score transformation. This involved subtracting the eigenvalue of each channel from its mean and dividing it by its standard deviation. The test set data were then normalized using the mean and standard deviation of the same channels in the training set. Finally, each electrode channel in the training set was transformed to signal motion data ${\;x}_{1},x_{2},x_{3},...,x_{i}.$ The mathematical formula is as follows:

(3) $$y_{i}=\frac{x_{i}-\mu }{\sigma }$$

The symbol μ represents the mean of the data for a single-electrode channel, and σ represents its standard deviation. The data were normalized sequentially over 12 channels for the EMG signals and over 36 channels for the acceleration signals. Z-score normalization is a process that normalizes the value of the true signal from 10^−4^ to approximately 1. This method preserves the distribution of the signal in each electrode channel, reduces the impact of outliers, and ensures that the signals have a similar scale across individuals and experimental conditions.

#### 2.3.3. Signal Segmentation

The experimental data signals were filtered to remove high-frequency noise and irrelevant signal components, retaining the main physiological signals. However, it is important to note that surface EMG and acceleration signals contain action and rest phases, and different actions or gestures correspond to different patterns of muscle activity. Signal segmentation allows for a clear definition of signals from different phases, aiding in the analysis of EMG signals within specific time windows and improving analysis accuracy and reproducibility [24]. Subsequent analysis of the signals at each stage can better meet study needs.

**Figure 3:**
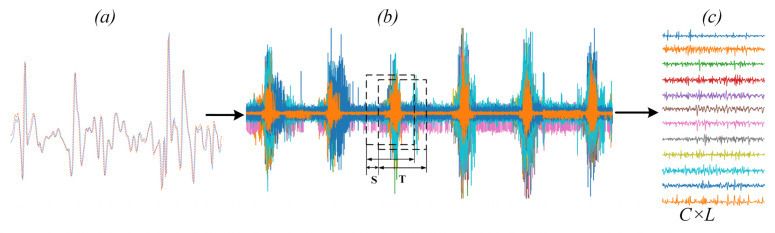
Pretreatment process. (**a**) Filtering and denoising. (**b**) Z-score normalization and signal segmentation. (**c**) Signal after preprocessing.

In order to satisfy the real-time nature of the HCI system, the delay of the system responding to user input should be as small as possible, and the control delay of the HCI control system should not exceed 300 ms [31]. The data were divided into sequences of equal length using a sliding window process. Labels were added to each sequencing sample to construct the time series samples. The duration of the selection window in this paper was T milliseconds, and it slid every S milliseconds. The selection of window length significantly affected the study’s outcomes. As there is no universal formula for the optimal window length, we made these choices based on previous research. In this paper, we set T to 100 ms, 150 ms, 200 ms, and 250 ms, and S to 50 ms, considering the existing studies [32,33,34]. Figure 3 illustrates the signal interception process, and the corresponding calculation formula is presented below:

(4) L=T⋅F/1000

(5) $$L_{d}=S\cdot F/1000$$

where L represents the length of the time window, $L_{d}$ represents the length of the sliding step, F is the sampling rate of the signal 2000 Hz. We obtained the data sample $x\in R^{C\times L}$, where C is the number of signal acquisition channels, each $L_{d}$ data point is captured, and the length of the captured window is L data points, resulting in a signal of size C×L.

### 2.4. Two-Stream Residual Network

**Figure 4:**
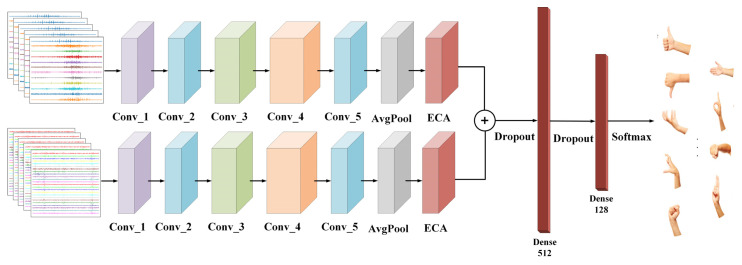
A two-stream residual gesture recognition network model based on attention mechanism.

This paper used a two-stream residual network model with an attention mechanism for multi-gesture recognition. Neural networks allow for end-to-end feature extraction. The network structure is depicted in Figure 4, and the public gesture dataset was utilized for the model to achieve the training optimization design of the model and to extract the end-to-end gesture recognition features comprehensively.

In the experiments, the network model was fed with the EMG and acceleration signals generated by the sliding window. The two branch networks used preprocessed EMG data $x\in R^{W_{1}\times H_{1}}$ and acceleration data $y\in R^{W_{2}\times H_{2}}$ as inputs. Here, $W_{1}$ and $H_{1}$ represent the width and height of the EMG signal x, while $W_{2}$ and $H_{2}$ represent the width and height of the acceleration signal y. The network model can convolve not only along the transverse direction to obtain the morphological information of the input signal but also along the longitudinal direction to obtain the spatial characteristics of the different electrode channels.

### 2.5. Residual Network

Deep convolutional neural networks have achieved significant advancements in the area of image recognition [35]. Research indicates that the depth of a model is a critical factor. Increasing the number of convolutional neural layers allows for the extraction of various levels of features. As the layers of the network model become deeper [36,37], the dimension of the extracted features increases, resulting in more information being obtained. However, in traditional deep neural networks, stacking multiple layers of the network results in a gradual decay of the signal as it propagates, causing the gradient to disappear [38]. Training the network becomes challenging, particularly in deeper network structures. As the network’s depth increases, the model’s accuracy saturates and then rapidly degrades.

**Figure 5:**
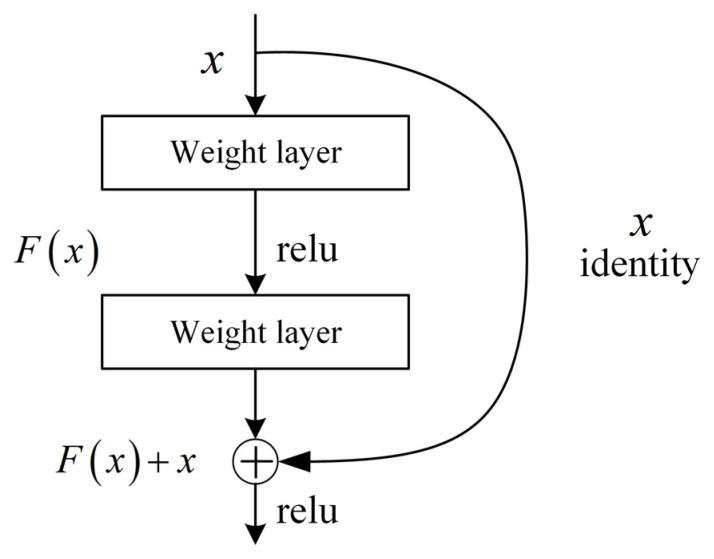
Residual block schematic diagram.

The emergence of deep residual networks has greatly reduced this phenomenon. This is because residual networks introduce a new structure that is more conducive to optimization convergence. The residual structure adds a short-circuit mechanism between every two layers to form a residual block, as shown in Figure 5.

The residual block offers the advantage of proposing two mapping methods: residual mapping and identity mapping. Residual mapping includes the convolutional layer, batch normalization layer, and activation function (ReLU), while identity mapping refers to the bypass connection in Figure 4, which forms a shortcut connection in the neural network. The residual mapping produces the output F(x), while the shortcut connection produces the output x. Therefore, the final output value of this residual block is y=F(x)+x.

In the backpropagation process, assuming the loss function is L, we had to calculate the gradient $\frac{\partial L}{\partial x}$ of the loss function with respect to the input x in order to update the network parameters. According to the chain rule of backpropagation, it could be obtained as follows:

(6) $$\frac{\partial L}{\partial x}=\frac{\partial L}{\partial y}\cdot \frac{\partial y}{\partial x}$$

where $\frac{\partial L}{\partial y}$ denotes the gradient of the loss function with respect to the output y, and $\frac{\partial y}{\partial x}$ denotes the gradient of the output y with respect to the input x. Since y=F(x)+x, $\frac{\partial y}{\partial x}$ can be expressed as $\frac{\partial y}{\partial x}=\frac{\partial F\left(x\right)}{\partial x}+\frac{\partial x}{\partial x}=\frac{\partial F(x)}{\partial x}+1$, where $\frac{\partial F\left(x\right)}{\partial x}$ is the gradient of the residual mapping $F\left(x\right)$ to the input x.

Since the residual mapping $F\left(x\right)$ is usually a shallow network, its gradient $\frac{\partial F\left(x\right)}{\partial x}$ is small or even close to zero. In contrast, the 1 in $\frac{\partial y}{\partial x}$ indicates the gradient of the constant mapping, which is always 1.

Therefore, even if $\frac{\partial F\left(x\right)}{\partial x}$ is small, $\frac{\partial y}{\partial x}$ can still maintain a large gradient due to the jump connections in the residual block, thus avoiding the gradient vanishing problem and allowing the gradient to propagate more smoothly to the shallower layers of the network. This residual function not only deepens the network’s layers to extract a richer feature set but also facilitates optimization and prevents gradient dispersion or explosion. Theoretically, as the depth of the network increases, the network should remain in optimal condition without any negative impact on performance [38]. Table 2 shows the structural parameters of ResNet-18.

### 2.6. ECA Attention Mechanism

**Figure 6:**
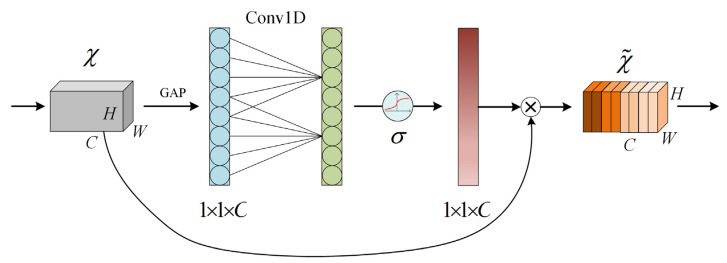
ECA mechanism.

The focus of an increasing number of researchers is on improving the performance and efficiency of deep neural networks, with the development of deep learning technology. Attentional mechanisms [39] have been shown to be an effective way to make networks pay more attention to important features and suppress unimportant ones. Attention mechanisms have been successfully applied in several fields, including natural language processing [40,41], computer vision [42], and speech recognition [43], by mimicking the way humans allocate their attention when processing information. The gradual application of attention mechanism to myoelectric gesture recognition has proven to be crucial in improving the performance of the recognition model. However, conventional attention mechanisms often demand significant computational resources and a large number of parameters, which can restrict their practicality. Therefore, this paper introduces the ECA (efficient channel attention) mechanism [44] in the network model construction. ECA-Net improves the SE-Net module [45] by proposing a local cross-channel interaction strategy without dimensionality reduction and adaptive selection of a one-dimensional convolutional kernel size, resulting in performance optimization. This paper introduces the ECA mechanism to assign weights to each channel’s features. This allows the network to focus on important features and fully learn key information from the data. The ECA module is illustrated in Figure 6.

The ECA mechanism prevents dimensionality reduction and effectively implements local cross-channel interactions using one-dimensional convolution to extract inter-channel dependencies. First, the input features undergo a global average pooling operation. Then, a one-dimensional convolution operation is performed using a convolution kernel size of k, which is determined by the number of channels and an adjustable hyperparameter. The weights of each channel are then obtained after applying a Sigmoid activation function. The final output feature representation is obtained by multiplying the weights with the corresponding elements of the original input features.

Assuming the input feature map is $\chi \in R^{W\times H\times C}$, where C represents the number of channels, and W and H represent the height and width of the feature map respectively, the channel attention weights are:

(7) $$\omega_{c}=\sigma \left(C1D_{k}\left(g\left(\chi \right)\right)\right),$$

where C1D stands for one-dimensional convolution involving only k parameter information, $g\left(\chi \right)$ stands for channel-wise global advection pooling (GAP), and σ represents the Sigmoid function. The term $g\left(\chi \right)$ refers to:

(8) $$g\left(\chi \right)=\frac{1}{WH}\sum_{i=1}^{W}\sum_{j=1}^{H}\chi_{ij},$$

Adaptive variation of the convolutional kernel size was used to perform one-dimensional convolutional operations based on the number of feature channels. The kernel size was determined by the formula $k=\left|\left(\log_{2}^{C}+\lambda \right)/\gamma \right|$, where λ and γ were set to 1 and 2, respectively, and C is the number of input feature channels. k is the adaptive one-dimensional convolution kernel size. The convolution step was set to 1.

The attention weights were used to weight the input features:

(9) $$Y_{c}=\omega_{c}\;\cdot \;\chi_{c},$$

where $Y_{c}$ is the weighted feature map and $\chi_{c}$ is the c-th channel of the input feature map. The final output feature map is the elemental sum of the weighted feature map as:

(10) $$Y=\sum_{C=1}^{C}Y_{c}$$

The ECA mechanism ensures performance results and model efficiency by capturing cross-channel information interactions.

## 3. Results

### 3.1. Experimental Parameter Settings

The experiment was conducted on a Windows 10 64-bit operating system with 16 GB of RAM. The model structure’s main body was built on the PyCharm platform using Tensorflow GPU version 2.6.0. The network training was accelerated using an GeForce RTX 3090 GPU (NVIDIA, Santa Clara, CA, USA). The epoch was set at 30, and the batch size was set to 256. To accelerate neural network training and prevent missing the optimal solution later due to a large learning rate, we set the initial learning rate to 0.001 and the weight decay factor to 0.0005. The learning rate was adjusted using the ReduceLROnPlateau method in Keras (version 2.6.0). This method reduces the learning rate when the loss value stops decreasing or the accuracy stops increasing. The factor was set to 0.1 and the patience was set to 1. Dropout methods were employed between fully connected layers to prevent overfitting.

The loss function employs the cross-entropy function and the error loss between the network prediction result and the true label of the sample is calculated as follows:

(11) $$loss=-\sum_{i=1}^{n}y_{i}\log\left(\overset{\wedge }{y_{i}}\right),$$

where $y_{i}$ represents the true value of the sample belonging to the *i*-th class of labeled gestures, $\overset{\wedge }{y_{i}}$ represents the predicted value of the sample belonging to the *i*-th class of labeled gestures, n denotes the number of classes, and loss represents the error between the predicted and true values of the sample. To enable loss calculation using cross-entropy, we chose labels that were uniquely thermally encoded. Meanwhile, the backpropagation algorithm efficiently computed the gradient of the cross-entropy loss function with respect to the network parameters. The Adam optimizer was then used to update the network weights, minimizing the loss function and improving the model’s performance on the training data. The network training hyperparameters are shown in Table 3.

### 3.2. Parametric Experiments

**Figure 7:**
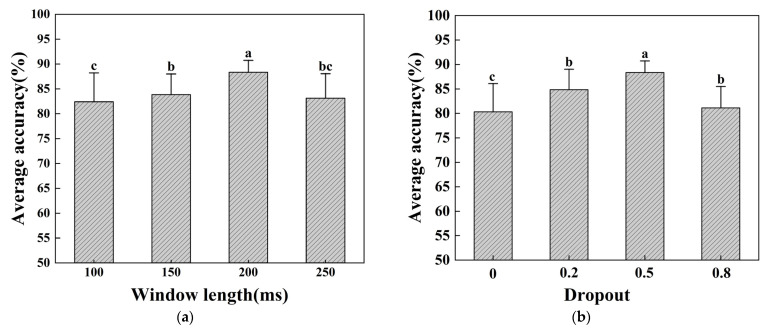
Average accuracy of gesture recognition for all subjects under different parameters: (**a**) average accuracy of gesture recognition with four different window lengths; (**b**) average accuracy of gesture recognition with different dropout rates. Different lowercase letters indicate a significant difference in statistical analysis across groups.

In practical applications, such as myoelectric prostheses and exoskeleton systems, it is crucial that myoelectric gesture recognition systems have a fast response time without any noticeable delays for the user. To achieve this, we conducted experiments with the network model parameters in our study to ensure the accurate and prompt recognition of gestures. Figure 7 demonstrate the impact of parameter selection on gesture recognition accuracy.

The length of the individual samples’ input into the network model is determined by the size of the time window, which refers to the number of time samples. When the time window is larger, there are more time samples; conversely, when it is smaller, there are fewer time samples. We set the sliding step size to 50 ms to intercept different lengths of time windows from the surface EMG signals and acceleration signals to evaluate the effect of different time window sizes on the accuracy of gesture recognition. The differences between the different window lengths were then statistically evaluated to select the optimal window length. The effect of the time window size on the average gesture recognition accuracy is shown in Figure 7a.

Figure 7a illustrates that the average accuracy of gesture recognition varied with different window lengths. When the time window was 100 ms or 150 ms, the average accuracy was low, but when the window length was 200 ms, the average recognition accuracy increased by approximately 5%. However, when the time window length reached 250 ms, the average accuracy decreased. In general, as the window length increases, the accuracy of gesture recognition shows a trend of increasing and then decreasing. Specifically, when the window length is short, the data may become too dispersed, which affects the model’s ability to capture gesture features, thus reducing the recognition accuracy. Conversely, when the window length is long, the data may become lost or confused, which also affects the accuracy of gesture recognition.

Figure 7b illustrates the impact of dropout on the accuracy of gesture recognition. Dropout is a widely used regularization technique that reduces overfitting by randomly dropping some of the connections between neurons during neural network training. Based on the experimental results presented in Figure 7b, it is evident that the effect was more pronounced when the dropout rate was set to 0.5.

Statistical evaluation of the differences between conditions can yield more comprehensive information. We performed ANOVA tests and found significant differences between the different window length conditions as well as between different dropouts, suggesting that choosing the right window length and dropout value is critical for improving the accuracy of gesture recognition. With further post-hoc comparisons, we were able to determine that a window of 200 ms length and a dropout of 0.5 had greater variance, and we selected them as the best parameters for optimizing gesture recognition performance.

### 3.3. Comparison Experiments

**Figure 8:**
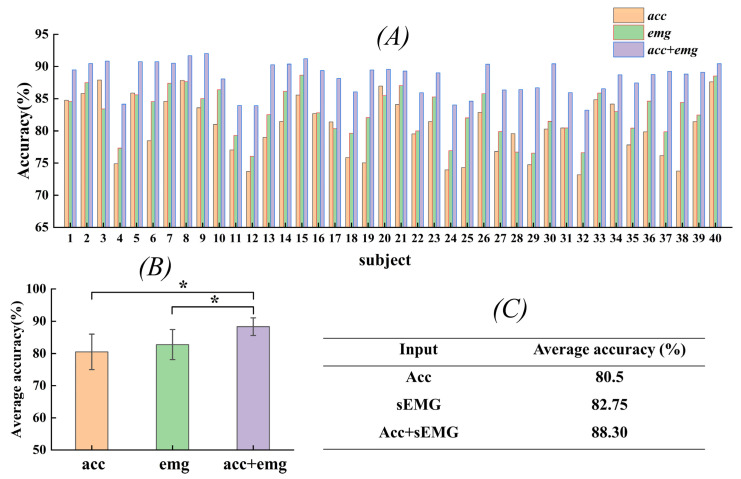
Accuracy of gesture recognition using different modal inputs in the DB2 database. (**A**) Gesture recognition accuracy for each subject. (**B**) Average gesture recognition accuracy after statistical analysis. (**C**) Values of average accuracy rate. * Indicates a significant difference in statistical analysis across groups.

Surface EMG signals and acceleration signals are two different types of modal information, and the contribution of multimodal information to gesture recognition can be evaluated by comparing the performance of single-stream and dual-stream networks. In order to verify the effectiveness of the two-stream residual network model constructed in this paper and the multi-information fusion method in the gesture recognition task, experiments were conducted on 40 subjects in the DB2 dataset to verify the effects. The results were compared with those of a single-stream residual neural network. Single-stream residual networks consider only one input, which can be either an EMG signal or an acceleration signal. Figure 8 displays the experimental results.

As can be seen in the experimental results in Figure 8A, the two-stream network achieved better results for each subject, which indicates that the two-stream residual network is able to capture the characteristics of human motion more comprehensively. Specifically, in the single-stream network, only one type of modal information is considered, e.g., only EMG signals or acceleration signals are used for gesture recognition. In contrast, in a two-stream network, two different types of modal information, i.e., EMG and acceleration signals, are combined, which enables the more comprehensive capture of gesture features. Additionally, performance differences were observed among subjects, which may have been influenced by individual characteristics and learning ability.

The average recognition results of this network model for three different inputs on the test set are shown in Figure 8B,C. The experimental results comparing the single-stream residual neural networks show that the average recognition accuracy values were about 8% and 6% higher than that of the EMG and acceleration signals. In addition, statistical analyses revealed significant differences between the two network models. This study demonstrates that information fusion enhances the network’s ability to accurately understand and recognize various gesture actions, leading to significant improvements in gesture recognition performance.

**Figure 9:**
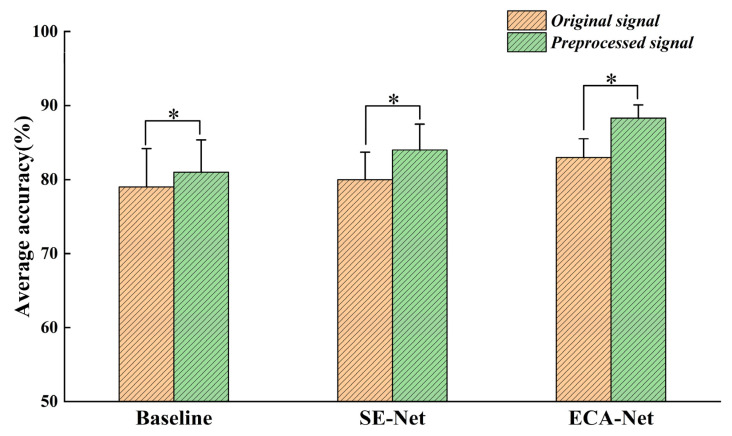
Effects of three different attentional mechanisms on average accuracy. * Indicates a significant difference in statistical analysis across groups.

Three sets of experiments were designed to verify the improvement of ECA-Net on model gesture recognition performance. The study consisted of three experimental groups: one without an attention mechanism, one with the addition of the SE-Net module, and one with the addition of the ECA-Net module. A simultaneous comparison was conducted using both raw and preprocessed data as the input signals. The results of the experiments are presented in Figure 9.

Regarding the results of the attention mechanism experiments, the shorter error line in Figure 9 indicates a smaller standard deviation, which represents more concentrated data. Statistical analysis revealed significant differences in accuracy between the raw and preprocessed data for each subject under the three conditions. Whether using raw data as input or after preprocessing, the performance of the gesture recognition model was significantly improved by adding the ECA-Net. On the other hand, the performance improvement after adding the SE-Net was slightly worse compared to the ECA-Net. This suggests that the ECA-Net is more suitable as an attention mechanism for gesture recognition tasks than SE-Net, and its stronger feature modeling capability allows the model to determine different gesture actions more accurately.

### 3.4. Average Performance Evaluation

To more fully assess the model’s ability to generalize, reliability, and stability, we took steps to reduce the influence of individual subjects on the results. We analyzed the data from all subjects together to ensure a more representative and generalized assessment of the model. By analyzing the data from all subjects as a whole, we obtained an average score for model performance, which allowed us to obtain more reliable and comprehensive statistical results that further deepened our understanding and assessment of model performance.

**Figure 10:**
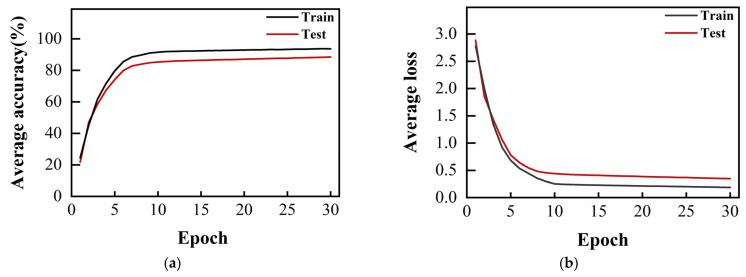
Mean accuracy and mean loss function curves for all subjects: (**a**) average accuracy for training and test sets; (**b**) average loss function of training and test sets.

In Figure 10, we present the average accuracy and loss function curves for 40 participants on the training and test sets. In the figure, we can observe that the average accuracy of the test set stabilized at around 88% during the training process, while the average loss function gradually converged, which further confirms the stability of the model and the reliability of its performance.

**Figure 11:**
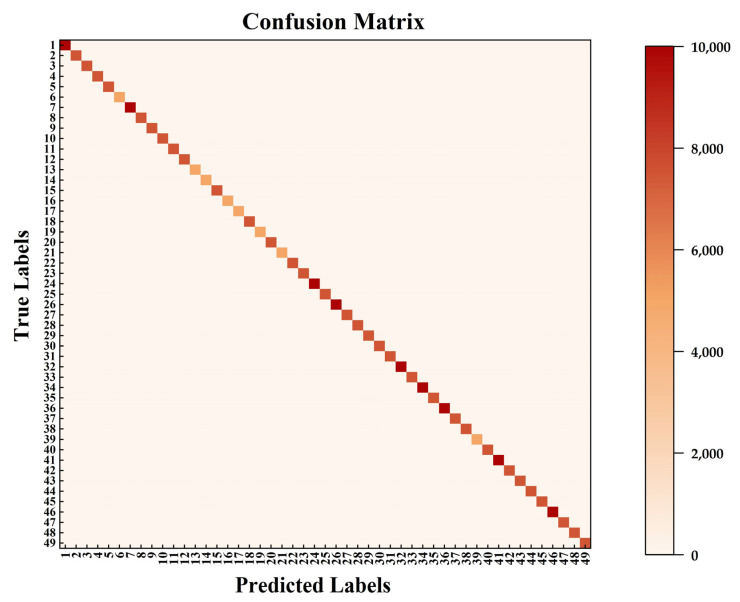
Heatmap of the mean confusion matrix for 49 gestures across all subjects.

A confusion matrix heat map is a way to visualize confusion matrix data. Figure 11 shows a heat map of the average confusion matrix for the 49 gestures containing all subjects. The plot is a square matrix with the predicted gesture labels plotted on the horizontal coordinate and the actual gesture labels plotted on the vertical coordinate. Each element in the confusion matrix is represented by a color, with different shades or tones of color representing different values. The darker the color, the higher the corresponding classification result. The cells on the main diagonal represent the number of samples correctly classified by the model, while the other cells represent the number of samples misclassified by the model. In the figure, it can be seen that the cells on the main diagonal are darker in color, indicating that the classified samples are mostly concentrated on the diagonal, which is a more intuitive reflection of the good classification performance of the network model in this paper.

### 3.5. Comparative Analysis with Other Methods

The method in this paper was compared with the models that have been studied on the NinaPro DB2 database in recent years. Among them, Ding et al. [22] proposed a parallel multiscale convolutional structure with a larger convolutional kernel size. The structure achieved a recognition accuracy of 78.86% for 17 gestures. Hu et al. [31] employed a hybrid attention-based architecture (CNN-RNN) to accurately recognize 49 gestures with an 82.2% success rate. Zhou et al. [26] proposed the multi-stream feature fusion network (MSFF-Net) model, which improved the accuracy to 87.02%. J.A. Sandoval-Espino et al. [46] conducted an experiment using CNNs to analyze four sets of the time-domain features of EMG signals. The results showed an accuracy of 87.56%. The results of different methods of identification are shown in Table 4.

## 4. Discussion

Gesture recognition is becoming increasingly important in human–computer interaction systems and has been studied by many researchers using various methods. Some studies have focused on signal processing. Geng et al. [21] proposed an approach using instantaneous sEMG images that obtained significant results on the DB2 dataset. Other studies focused on feature extraction, J.A. Sandoval-Espino et al. [46] extracted four features in the time domain of the EMG signal for input into the CNN. Other studies have employed pattern recognition methods. Hu et al. [31] utilized a CNN-RNN architecture, while Ding [22] implemented a CNN model with a larger convolutional kernel.

It can be stated that the preprocessing, network structure, and optimization parameters play a pivotal role in the analysis of surface electromyographic signal (sEMG) data using deep neural networks [38]. The appropriate selection and tuning of these factors can result in enhanced model accuracy, accelerated convergence, and a notable improvement in model performance and effectiveness. Additionally, the study of hand posture synergy is of significant importance for research in the field of rehabilitation and hand motor control, as it represents one of the principal methods for the understanding of the characteristics and patterns of hand movements [47,48,49]. It aims to analyze the relationship between different hand postures in EMG signals and to construct a prediction framework capable of predicting and modeling hand postures.

The myoelectric gesture recognition system needs to be real-time and accurate in practical applications. In our study, we experimented with the network model parameters and found the optimal time window length to be 200 ms and the dropout size to be 0.5. This suggests that the delay time is small and meets the requirements for human–computer interaction and other aspects. To test the effectiveness of the two-stream residual network and multimodal information fusion in gesture recognition, we input EMG signals and acceleration signals from 40 subjects into the single-stream residual network. The experimental results showed 6% and 8% increases in accuracy compared to the single-stream EMG input and single-stream acceleration signal input, respectively. This indicates that our method of fusing multiple signal sources is effective in improving gesture recognition accuracy. In the attention mechanism experiments, we input the original signal and the preprocessed signal into three network models. The network model with the addition of the ECA-Net outperformed the other two models in both the original and processed signals, demonstrating the advantage of the ECA mechanism. To minimize individual subject variability, we computed the mean performance across all subjects. We verified the generalizability of our model by analyzing the average accuracy, loss function, and confusion matrix.

In summary, this study presents a thorough evaluation of the model’s performance by analyzing experimental data on gesture recognition from 40 subjects. The experimental results indicate that the two-stream residual network model constructed in this paper has a significant advantage in the gesture recognition task for large-scale subjects, and its performance is stable. Future studies could further explore its inter-subject variability and further optimize the performance of the gesture recognition model.

## 5. Conclusions

In this study, we present a new approach for recognizing hand gestures using a two-stream residual network structure that fully utilizes multi-source information. One branch input is the electromyographic signal, and the other branch input is the hand acceleration signal. Before inputting the signal, a series of preprocessing operations are performed, including noise reduction, normalization, and signal segmentation. These operations aid in extracting effective features. The residual network facilitates information transfer and gradient flow. The network model in this paper can extract features by convolving along the transverse time domain and the longitudinal direction to obtain spatial features of different electrode channels. The ECA mechanism was introduced to enable the network to automatically learn and pay attention to important features. This enhances the network’s recognition ability and robustness. The experimental results indicate that the method presented in this paper exhibits reliable stability and high accuracy in performance. It provides a new feasible scheme for multi-classification gesture recognition.

## Figures and Tables

**Table 1:** Grouping of DB2 dataset labels.

Label	Gesture Classification
0	Rest
1~17	Basic movements of fingers
18~39	Grasping and functional movements
40~49	Force patterns

**Table 2:** The structure of ResNet-18.

Layer Name	Output Size	Layer-18 Parameters
conv1	112 × 112	7 × 7, 64, stride 2 3 × 3 max pool, stride 2
conv2_x	56 × 56	$\left[\begin{array}{l} 3\times 3,64\\ 3\times 3,64 \end{array}\right]\times 2$
conv3_x	28 × 28	$\left[\begin{array}{l} 3\times 3,128\\ 3\times 3,128 \end{array}\right]\times 2$
conv4_x	14 × 14	$\left[\begin{array}{l} 3\times 3,256\\ 3\times 3,256 \end{array}\right]\times 2$
conv5_x	7 × 7	$\left[\begin{array}{l} 3\times 3,512\\ 3\times 3,512 \end{array}\right]\times 2$
output layer	1 × 1	average pool, fc, softmax

**Table 3:** Training hyperparameters.

Hyperparameter	Numerical Value
Epoch	30
Batch size	256
Optimizer	Adam
Initial learning rate	0.001
Weight attenuation factor	0.0005

**Table 4:** Comparison of different gesture recognition methods.

Author	Dataset	Category	Classifier	Accuracy (%)
Ding	DB2	17	CNN	78.86
Hu	DB2	50	CNN-RNN	82.2
Zhou	DB2	49	MSFF-Net	87.02
J.A.	DB2	50	CNN	87.56
Our work	DB2	49	TS-ResNet	88.3

## Data Availability

Publicly available dataset NinaPro DB2 database was analyzed in this study. Data can be found here: http://ninapro.hevs.ch, accessed on 19 March 2024.
